# Antibody-Based In Vivo PET Imaging Detects Amyloid-β Reduction in Alzheimer Transgenic Mice After BACE-1 Inhibition

**DOI:** 10.2967/jnumed.118.213140

**Published:** 2018-12

**Authors:** Silvio R. Meier, Stina Syvänen, Greta Hultqvist, Xiaotian T. Fang, Sahar Roshanbin, Lars Lannfelt, Ulf Neumann, Dag Sehlin

**Affiliations:** 1 Department of Public Health and Caring Sciences/Geriatrics, Uppsala University, Uppsala, Sweden; 2 Department of Pharmaceutical Biosciences, Uppsala University, Uppsala, Sweden; 3 BioArctic AB, Stockholm, Sweden; and; 4 Neuroscience Research, Novartis Institutes for BioMedical Research, Basel, Switzerland

**Keywords:** Alzheimer’s disease, positron emission tomography (PET), antibody-based radioligand, BACE-1 inhibitor NB-360, amyloid-β

## Abstract

Visualization of amyloid-β (Aβ) pathology with PET has become an important tool for making a specific clinical diagnosis of Alzheimer disease (AD). However, the available amyloid PET radioligands, such as ^11^C-Pittsburgh compound B, reflect levels of insoluble Aβ plaques but do not capture soluble and protofibrillar Aβ forms. Furthermore, the plaque load appears to be fairly static during clinical stages of AD and may not be affected by Aβ-reducing treatments. The aim of the present study was to investigate whether a novel PET radioligand based on an antibody directed toward soluble aggregates of Aβ can be used to detect changes in Aβ levels during disease progression and after treatment with a β-secretase (BACE-1) inhibitor. **Methods:** One set of transgenic mice (tg-ArcSwe, a model of Aβ pathology) aged between 7 and 16 mo underwent PET with the Aβ protofibril–selective radioligand ^124^I-RmAb158-scFv8D3 (where RmAb is recombinant mouse monoclonal antibody and scFv is single-chain variable fragment) to follow progression of Aβ pathology in the brain. A second set of tg-ArcSwe mice, aged 10 mo, were treated with the BACE-1 inhibitor NB-360 for 3 mo and compared with an untreated control group. A third set of tg-ArcSwe mice, also aged 10 mo, underwent PET as a baseline group. Brain tissue was isolated after PET to determine levels of Aβ by ELISA and immunohistochemistry. **Results:** The concentration of ^124^I-RmAb158-scFv8D3, as measured in vivo with PET, increased with age and corresponded well with the ex vivo autoradiography and Aβ immunohistochemistry results. Mice treated with NB-360 showed significantly lower in vivo PET signals than untreated animals and were similar to the baseline animals. The decreased ^124^I-RmAb158-scFv8D3 concentrations in NB-360–treated mice, as quantified with PET, corresponded well with the decreased Aβ levels measured in postmortem brain. **Conclusion:** Several treatments for AD are in phase 2 and 3 clinical trials, but the possibility of studying treatment effects in vivo on the important, nonfibrillar, forms of Aβ is limited. This study demonstrated the ability of the Aβ protofibril–selective radioligand ^124^I-RmAb158-scFv8D3 to follow disease progression and detect treatment effects with PET imaging in tg-ArcSwe mice.

Alzheimer disease (AD) is the most common neurodegenerative disease, and the aging of the population will increase the number of people affected. However, no treatment is yet available to halt the pathologic changes underlying disease progression. The amyloid hypothesis (1) states that aggregation of the amyloid-β (Aβ) protein eventually leads to neurodegeneration and finally to dementia. To reduce Aβ pathology, immunotherapy studies have been conducted on several Aβ antibodies (2). Aducanumab, which targets Aβ aggregates, recently showed benefits in AD patients (3), and BAN2401 (4), the humanized version of mAb158 (5,6), which targets soluble Aβ protofibrils, is in a phase 2b clinical trial. In addition, several low-molecular-weight β-secretase (BACE-1) inhibitors, aimed at reducing Aβ production, are in clinical trials.

Because the pathologic Aβ accumulation occurs over many years, there is a crucial need for sensitive, specific, and representative diagnostic tools to follow disease progression and the effects of disease-modifying treatments in clinical studies.

Several small-molecule PET ligands, such as ^11^C-Pittsburgh compound B (PiB) and ^18^F-florbetaben, visualize amyloid plaques and have become important tools in AD diagnostics. However, amyloid PET may be positive years before any clinical symptoms appear (7) and shows an early saturation during disease progression (8). In addition, diagnosis with ^11^C-PiB and analogs has limited value when the Aβ pathology is diffuse, because diffuse plaques largely lack the β-sheet fibrillar structure to which these radioligands bind (9). Studies on Aβ toxicity have indicated that soluble aggregates, such as oligomers and protofibrils, are the most neurotoxic Aβ species (10–12). In addition, the brain level of soluble Aβ protofibrils seems to be a more dynamic indicator of disease severity and may thus be a better marker for disease progression than insoluble plaques (8,11). It is also likely that novel treatments directed at decreasing Aβ production (β-secretase inhibitors) or enhancing Aβ clearance (immunotherapy) will reduce levels of soluble Aβ before an effect on plaque load can be detected. Treatment effects could thus be difficult to monitor with the current amyloid PET tracers. In fact, a recent study found only subtle changes in amyloid with the PET tracer ^18^F-florbetapir in APPPS1-21 mice treated with a BACE-1 inhibitor (JNJ-49146981) for 12 mo (13). Hence, a PET radioligand that visualizes forms other than insoluble, fibrillar Aβ could be an important tool for detecting drug effects in clinical trials.

We previously described the radioligand ^124^I-RmAb158-scFv8D3 (where RmAb is recombinant mouse monoclonal antibody and scFv is single-chain variable fragment), which is based on mAb158, that binds selectively to soluble Aβ protofibrils, with moderate and low cross-reactivity to Aβ fibrils and monomers, respectively (6,14,15). Antibodies, because of their large molecular size, are generally characterized by limited passage across the blood–brain barrier. To enhance brain uptake of mAb158 and enable its use as a PET radioligand, it was functionalized with 2 single-chain variable fragments of the transferrin receptor antibody 8D3 (16). This conjugation leads to active transcytosis via the transferrin receptor into the brain (17). Several similar constructs (15,18,19) have been used to image the progressive accumulation of soluble Aβ aggregates in transgenic mice harboring the Arctic (Aβ precursor protein [AβPP] AβE693G) and the Swedish (AβPP KM670/671NL) *AβPP* mutations (tg-ArcSwe mice (20)) at different ages, demonstrating the potential of bispecific antibodies as neuro-PET radioligands.

The aim of the present study was, in a first step, to investigate the ability of ^124^I-RmAb158-scFv8D3 to follow disease progression, that is, escalating brain Aβ pathology, in the tg-ArcSwe mouse model. To achieve this aim, in vivo ^124^I-RmAb158-scFv8D3 PET in tg-ArcSwe mice of different ages was compared with ex vivo measurement of brain radioactivity and Aβ pathology in postmortem analyzed brain tissue from the same mice.

In a second set of mice, Aβ reduction after treatment with the BACE-1 inhibitor NB-360 (21) was studied to investigate the ability of ^124^I-RmAb158-scFv8D3 to image the reverse effect, that is, a decrease in brain Aβ pathology. In previous studies, NB-360 has shown a robust Aβ reduction and a promising pharmacokinetic profile in AβPP transgenic mice. In addition, NB-360 has demonstrated a beneficial effect on cellular, long-range circuitry and memory in APP23xPS45 transgenic mice (22). Thus, in tg-ArcSwe mice we conducted a preclinical NB-360 treatment study, designed to resemble a clinical study of a new drug candidate, using ^124^I-RmAb158-scFv8D3 PET imaging to quantify the treatment effect in vivo.

## MATERIALS AND METHODS

### Animals

tg-ArcSwe mice (20) were scanned with PET to study disease progression at the age of 7, 10, 13, and 16 mo. The effect of NB-360 treatment was investigated in mice aged 10 mo at the start of the treatment, that is, at an age characterized by moderate Aβ pathology (23) but no detectable ^11^C-PiB PET signal (15). The number of mice in each age group is displayed in Supplemental Table 1 (supplemental materials are available at http://jnm.snmjournals.org).

Throughout the experiment, the animals were kept with free access to food and water in rooms with controlled temperature and humidity in an animal facility at Uppsala University. All experiments were approved by the Uppsala County Animal Ethics board (approval C17/14) following the rules and regulations of the Swedish Animal Welfare Agency and complied with the European Communities Council Directive of September 22, 2010.

### Radioligand

The recently described radioligand ^124^I-RmAb158-scFv8D3 was used for antibody-based PET imaging. Its expression and pharmacokinetics were described by Hultqvist et al. in 2017 (17). The radioligand is based on the well-studied antibody mAb158, which displays a selective binding to Aβ protofibrils (6,14,15). To increase the transport of mAb158 across the blood–brain barrier into the brain, 2 single-chain variable fragments of the transferrin receptor antibody 8D3 (16) were attached using short linkers to the C termini of the light chains of mAb158. This step enables monovalent transferrin receptor binding, which leads to efficient transcytosis over the blood–brain barrier. RmAb158-scFv8D3 was recombinantly expressed in Expi293 cells and purified as described previously (17).

### Radiochemistry

RmAb158-scFv8D3 was labeled with ^124^I using direct radioiodination (24). Radiolabeling was done in 6 batches; a 119 ± 31.3 MBq ^124^I solution (PerkinElmer Inc.) was preincubated for 15 min with NaI at a concentration of 10 μM. RmAb158*-*scFv8D3 was mixed with phosphate-buffered saline (PBS) and added to the iodine solution at 0.6 μg/MBq to a final volume of 380 μL. The reaction was activated by addition of 40 μL of chloramine-T solution (1 mg/mL) and quenched after 120 s by addition of 80 μL of sodium metabisulfite solution (1 mg/mL). The radiolabeled protein was purified of free iodine and low-molecular-weight components with a disposable NAP-5 size-exclusion column with a molecular-weight cutoff of 5 kDa (GE Healthcare). The high-molecular-weight fraction containing the labeled protein was eluted with 1 mL of PBS.

### PET Imaging

PET imaging was performed with ^124^I-RmAb158-scFv8D3 using a Triumph Trimodality System (TriFoil Imaging, Inc.). To reduce thyroidal uptake of ^124^I, the mice were given 0.5% NaI in the drinking water 1 d before radioligand injection and then 0.2% NaI until the PET scan. The mice in the disease-progression investigation were intravenously injected with 14.8 ± 2.6 MBq of ^124^I-RmAb158-scFv8D3 with a specific activity of 234 MBq/nmol and scanned 4 d afterward. The mice in the NB-360 treatment experiment were intravenously injected with 7.4 ± 1.3 MBq of ^124^I-RmAb158-scFv8D3 with a specific activity of 161 ± 12.7 MBq/nmol and scanned 6 d afterward. Before undergoing PET, the animals were anesthetized with 2.5% isoflurane, moved to the heated scanner bed, and kept anesthetized with 1.5%–2% isoflurane during the scan. The duration of the scan was 60 min, followed by a CT examination of 3 min (field of view, 8.0 cm).

Directly after scanning, the mice underwent a 2-min intracardiac perfusion with 0.9% NaCl. Brain and blood samples were collected, and ex vivo radioactivity was measured with a well counter (GE Healthcare).

PET data were reconstructed using 3-dimensional ordered-subsets expectation maximization (20 iterations). CT raw files were reconstructed using filtered backprojection. All subsequent processing of the PET and CT images was performed using the imaging software Amide, version 1.0.4 (25). The CT scans were manually aligned with a T2-weighted, MRI-based mouse brain atlas (26) containing outlined regions of interest for whole brain, cortex, and hippocampus. The PET images were then aligned with the CT images, and the atlas and PET data could be quantified in regions of interest.

### Ex Vivo Autoradiography

Frozen right hemispheres of ^125^I-RmAb158-scFv8D3–injected mice were cryosectioned (20 μm) with a Leica CM 1850 at −25°C. Sections were stored at −20°C until use. Fresh tissue sections were placed in an x-ray cassette and exposed to positron-sensitive phosphor screens (MultiSensitive; PerkinElmer) for 6 d. The plates were then scanned with a Cyclone Plus Imager system (Perkin Elmer) at a resolution of 600 dpi. Images were analyzed by ImageJ software, version 1.51.

### NB-360 Treatment

**FIGURE 1.**
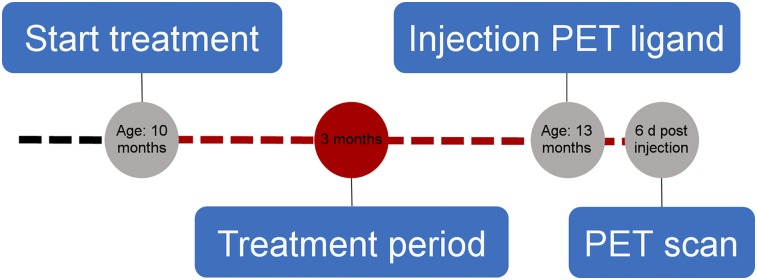
Overview of treatment and PET imaging. At age of 10 mo, animals were given either food containing BACE-1 inhibitor NB-360 or control food. At age of 13 mo, animals were injected with radioligand and were scanned with PET 6 d later. A third group was scanned with PET and analyzed at baseline age of 10 mo for comparison at starting point.

Three groups of mice were investigated (Fig. 1; Supplemental Table 1). A NB-360–treated group was provided food containing the BACE-1 inhibitor NB-360 (Novartis) at a concentration of 0.5 g/kg for 3 mo, whereas a vehicle group was maintained on control food until the age of 13 mo. Mice from 4 litters were randomly allocated to the NB-360– or vehicle-treated groups, and all mice were studied during the same period. A baseline group, included to provide an estimate of the Aβ pathology level before treatment, had free access to control food until the age of 10 mo, when PET scans and analyses were conducted. All groups were scanned on the same occasion.

### Brain Sample Preparation

After perfusion, brains were removed and dissected in half. The right hemisphere was either fixed in 4% PFA and embedded in paraffin or frozen on dry ice for later autoradiography analysis. The left hemisphere was immediately frozen on dry ice for further extraction and measurement of Aβ concentrations, as previously described (*15,26*). In short, brain tissue was homogenized with a tissue grinder (Teflon [The Chemours Co.] pestle), 2 × 10 strokes on ice, at a 1:5 weight-to-volume ratio in Tris-buffered saline (TBS) with cOmplete Protease Inhibitor Cocktail Tablets (Roche). A 250-μL volume of the homogenate was mixed with another 250 μL of TBS and centrifuged for 1 h at 16,000*g*. The supernatants were stored at −80°C until analysis. For extraction of TBS insoluble proteins, 27 μL of the original extract and 73 μL of 96% formic acid (FA) for an FA concentration of 70% were mixed for 30 s with a Kimble Pestle pellet grinder (Sigma-Aldrich) followed by 1 h of centrifugation at 16,000*g*. Supernatants were stored at −80°C until analysis.

### Biochemical Aβ Analyses

To break Aβ aggregates for better detection (*27*), TBS extracts from all transgenic mice were supplemented with 1% sodium dodecyl sulfate and kept for 5 min at 95°C in a compact dry heat block (ThermoFisher) and then further diluted to 0.05% sodium dodecyl sulfate.

Prepared samples were analyzed with a V-PLEX Aβ Peptide Panel 1 Kit (Meso Scale Diagnostics) for Aβ38, Aβ40, and Aβ42 using neoepitope-specific antibodies to the different Aβ C termini in combination with 6E10, which binds near the Aβ N terminus. The assay was conducted according to the user’s manual, and the plates were read using a Meso Scale Diagnostics imager.

Aβ protofibrils and oligomers were measured with a homogeneous ELISA, using the Aβ N terminus–specific 82E1 (IBL International/Tecan Trading AG) as both capture and detection antibody and a calibration curve of synthetic Aβ protofibrils for quantification (*18*). A 96-well half-area plate was coated overnight with 12.5 ng of 82E1 per well, followed by blocking with 1% bovine serum albumin in PBS. TBS brain extracts were diluted 1:25 and incubated overnight at 4°C. Soluble Aβ aggregates with a size of at least a dimer were then detected with biotinylated 82E1 (0.25 μg/mL) and streptavidin–horseradish peroxidase (Mabtech AB), diluted 1:4,000. Signals were developed with K blue aqueous tetramethylbenzidine substrate (Neogen Corp.) and read with a spectrophotometer at 450 nm.

For ELISA measurement of total Aβx-40 and Aβx-42, 96-well plates were coated overnight with 100 ng per well of polyclonal rabbit anti-Aβ40 or anti-Aβ42 (Agrisera) and blocked with 1% bovine serum albumin in PBS. FA-soluble brain extracts were neutralized with 2 M Tris and diluted 1:10,000 for Aβ40 and 1:750 for Aβ42 analysis and then were incubated overnight at 4°C. After incubation with biotinylated 6E10 (Nordic BioSite) (0.5 μg/mL) and streptavidin–horseradish peroxidase (Mabtech AB), diluted 1:5,000, signals were developed and read as above. All sample and secondary antibody dilutions were made in ELISA incubation buffer (0.1% bovine serum albumin, 0.05% polysorbate 20 in PBS).

### Immunohistochemistry

Five-micrometer sections from fixed, paraffin-embedded right-brain hemispheres were processed with an electronic rotary microtome (Hm340E; Thermofisher). Sections were deparaffinized and incubated in 3% H_2_O_2_ and 10% methanol in water for 15 min. Nonspecific binding was blocked with 3% bovine serum albumin in PBS-polysorbate 20 (0.1%) for 1 h and incubated overnight with a polyclonal rabbit-anti-Aβ40 antibody (Agrisera). Sections were incubated with biotinylated goat anti-rabbit antibody (Vector Laboratories Inc.) for 2 h at room temperature, followed by PBS washes and a 45-min incubation with avidin/biotin complex (Vector Laboratories). The staining was visualized by 3-min 3,3′-diaminobenzidine development and mounted with DPX mounting medium (a mixture of distyrene, tricresyl phosphate and xylene; Sigma-Aldrich). Pictures were captured with a DXM1200F microscope (Nikon Instruments Inc.) at a magnification of ×5 and further processed with Photoshop CC photomerge (Adobe) to a whole-brain panorama.

For Aβ and glial fibrillary acidic protein (GFAP) immunofluorescence double staining, sections were deparaffinized, underwent antigen retrieval in citrate buffer at 86°C for 20 min, and were further permeabilized and blocked for 1 h with 5% normal goat serum and 0.3% Triton X-100 (The Dow Chemical Co.) in PBS. Aβ and GFAP were stained with 6E10 (Nordic BioSite) and anti-GFAP (Dako), respectively. Alexa Fluor 488 anti-mouse and Alexa Fluor 555 anti-rabbit (Life Technologies) were used as secondary antibodies. Pictures were captured with an LSM700 confocal laser scanning microscope (Zeiss), and the software Zen 2012 was used for image processing.

### Statistics

All statistical analyses and graphs were done in Prism, version 6 (GraphPad Software, Inc.). Group results are reported as mean ± SD. Data were analyzed with 1-way ANOVA followed by the Bonferroni post hoc test.

## RESULTS

**FIGURE 2.**
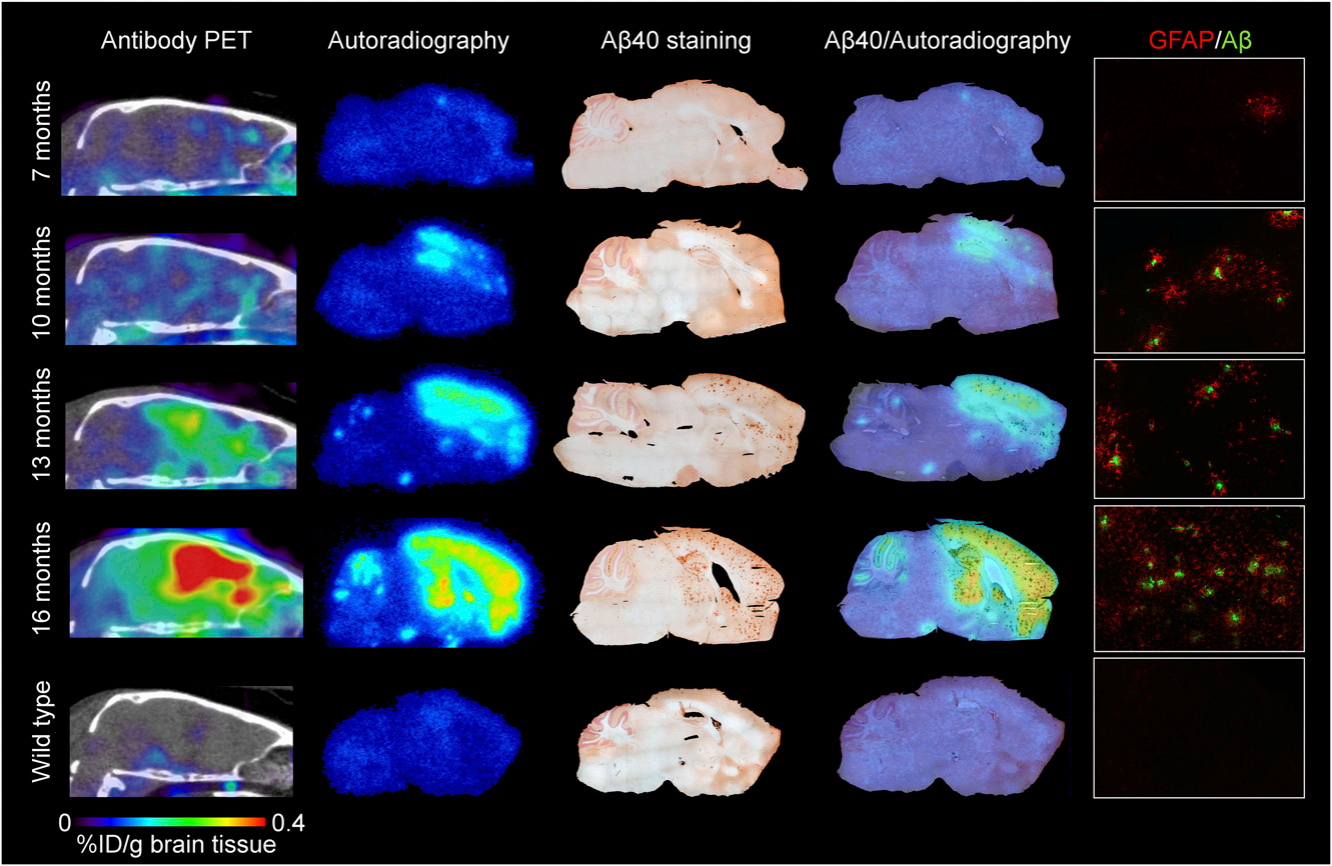
Disease progression and comparison between PET and ex vivo analysis. Representative images show disease progression in tg-ArcSwe mice from age of 7 mo up to 16 mo. From left to right, in vivo PET images with ^124^I-RmAb158-scFv8D3 are compared with corresponding ex vivo autoradiography and Aβ40 staining of same individual. Overlay of Aβ40 staining and ex vivo autoradiography highlights colocalization of injected ^124^I-RmAb158-scFv8D3 and Aβ40 pathology. GFAP/Aβ column shows activated astrocytes around Aβ deposits at ×20 magnification in cortex. %ID = percentage injected dose.

tg-ArcSwe mice between 7 and 16 mo old were scanned using ^124^I-RmAb158*-*scFv8D3 PET to study the ability of the radioligand to visualize progression of Aβ brain pathology. PET images corresponded well with ex vivo autoradiography images of brain sections prepared from perfused brain—that is, tissue devoid of any background signal from blood (Fig. 2). Aβ40 staining displayed a clear increase in Aβ burden with age. Further, the overlay of the autoradiography and the Aβ40 staining showed a high degree of colocalization between areas with high Aβ40 pathology and high retention of ^124^I-RmAb158*-*scFv8D3 (Fig. 2).

At the age of 7 mo, astrocyte activation measured with GFAP immunofluorescence was rare and limited to certain spots in the cortex. With increasing plaque load at the ages of 10 and 13 mo, astrocytes were activated around plaques. At the age of 16 mo, a comprehensive, areawide activation could be observed (Fig. 2).

**FIGURE 3.**
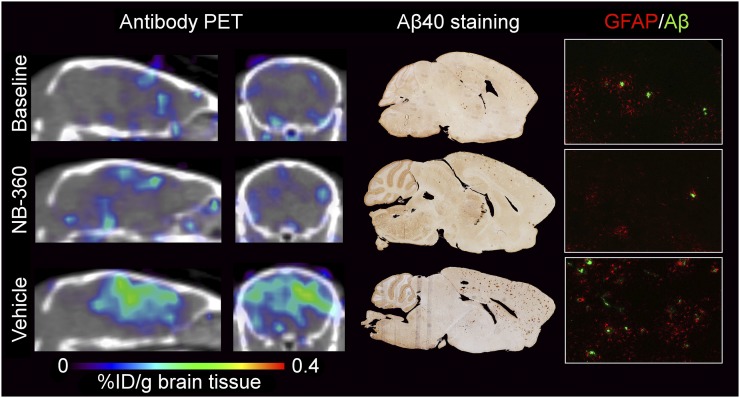
Overview of PET imaging, Aβ, and GFAP pathology after NB-360 treatment. Comparison is made of 3 groups of mice: baseline, NB-360, and vehicle. Sagittal and coronal PET images are at left, with brain concentrations of ^124^I-RmAb158-scFv8D3 expressed as percentage injected dose (%ID) per gram of brain tissue. Corresponding Aβ40-stained whole-brain images show total Aβ burden, including plaques. Aβ and GFAP staining demonstrates colocalization of Aβ deposits and reactive astrocytes at ×20 magnification.

In the second set of mice, PET scans were obtained after 3 mo of treatment with NB-360–containing food pellets or control food. A group of younger mice, representing the baseline, was also studied. ^124^I-RmAb158-scFv8D3 brain retention in the baseline group and the NB-360–treated group was low, and there was no notable difference between these groups, indicating that progression of Aβ pathology was halted by BACE-1 inhibitor treatment. In contrast, the group that had received control food showed a high PET signal in areas of abundant Aβ pathology (Fig. 3).

In line with the in vivo results, whole-brain Aβ staining revealed a higher Aβ burden in the vehicle group than in the NB-360 or baseline group. Pathology was observed mainly in the cortex, the hippocampus, and, with further disease progression in the vehicle group, the thalamus (Fig. 3). Aβ/GFAP double staining of the prefrontal cortex showed higher GFAP activity in the vehicle group than in the NB-360 or baseline group. Higher activation could especially be observed around plaques (Fig. 3).

**FIGURE 4.**
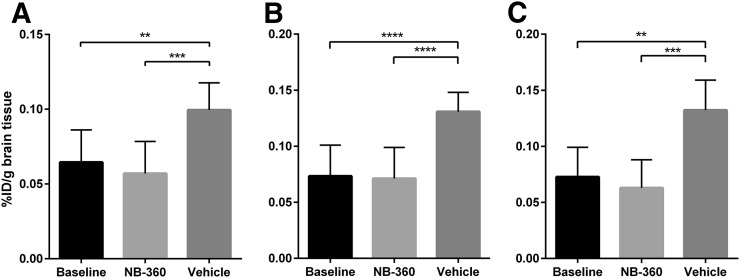
PET quantification of different brain regions. Image-based radioactivity is expressed as percentage injected dose (%ID) per gram of brain tissue, for whole brain (A), cortex (B), and hippocampus (C). While there was no significant difference between baseline and NB-360, vehicle group showed significantly higher activity in all brain regions (*P* = 0.002 in whole brain, *P* < 0.0001 in cortex, and *P* < 0.0001 in hippocampus). Values represent group mean with SD. **P < 0.01. ***P < 0.001. ****P < 0.0001.

**FIGURE 5.**
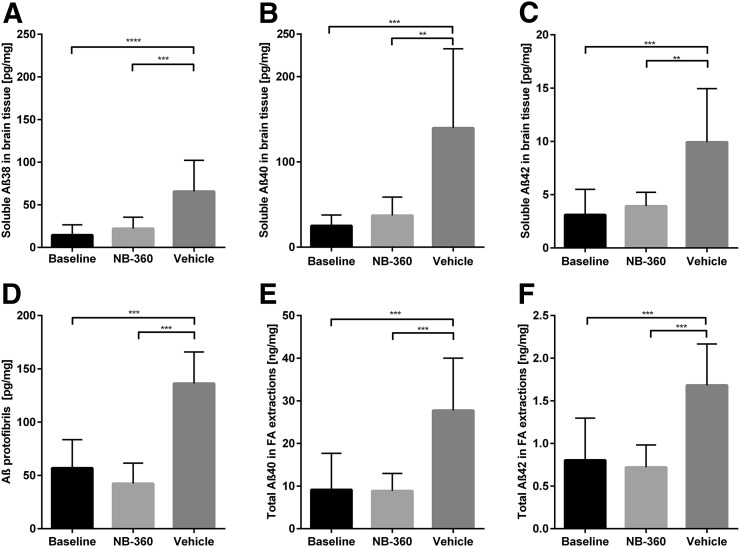
Aβ analysis in postmortem brain. (A–C) TBS-soluble Aβ38 (A), Aβ40 (B), and Aβ42 (C) in brain extracts from mice after PET scanning. Bars show group mean and SD. Baseline (*n* = 9) and NB-360–treated animals (*n* = 9) showed significantly lower levels of all 3 Aβ species than did vehicle animals (*n* = 11; *P* < 0.001). (D) Similar pattern was revealed with Aβ oligomer/protofibril–specific ELISA, showing significant difference between NB-360 and vehicle animals (*P* < 0.0001). (E and F) Total Aβ40 (E) and Aβ42 (F) load, measured in FA brain extracts, showed similar results. **P < 0.01. ***P < 0.001. ****P < 0.0001.

Image-based quantification of radioactivity showed that there was no significant difference in any of the studied regions between the baseline and NB-360–treated groups, whereas the vehicle group showed significantly higher activity in all brain regions, most pronounced in the cortex and hippocampus (Fig. 4; *P* = 0.002 in whole brain, *P* < 0.0001 in cortex, and *P* < 0.0001 in hippocampus). To verify the PET results, Aβ levels were biochemically assessed with an electrochemiluminescence immunoassay (MSD Technology Platform; Meso Scale Diagnostics) and ELISA in TBS-soluble and -insoluble brain extracts from the same mice that had undergone PET. There was no significant difference in TBS-soluble Aβ38, Aβ40, or Aβ42 levels between the NB-360 and baseline groups. However, a small trend toward slightly higher levels of all studied Aβ species could be observed in the NB-360 group (Figs. 5A–5C). The vehicle group showed significantly higher levels of soluble Aβ38, Aβ40, and Aβ42 than the baseline or treated animals (*P* = 0.0001, *P* = 0.0013, and *P* = 0.0001, respectively) (Figs. 5A–5C). Similar results were observed for soluble Aβ aggregates (Fig. 5D), with untreated mice displaying significantly higher levels than treated (*P* < 0.0001) or baseline (*P* < 0.0001) mice. Here, a trend was observed toward lower levels in the NB-360 group than in the baseline group. A ratio of soluble Aβ aggregates over total soluble Aβ displayed a nearly significant difference (*P* = 0.051) between the baseline group (1.9 ± 1.4) and the NB-360 group (0.8 ± 0.5), indicating a relative decrease in Aβ oligomerization as a result of BACE-1 inhibition.

ELISA analysis of FA-soluble brain extracts, which represent total Aβ40 and Aβ42, including plaques, also showed similar results. Aβ levels in baseline and NB-360–treated mice were equally low, indicating a similar plaque load in treated and baseline mice. Again, the vehicle group had significantly higher Aβ levels (for Aβ40, *P* < 0.0001, and for Aβ42, *P* < 0.0001) (Figs. 5E–5F).

## DISCUSSION

During the past decade, amyloid PET imaging, such as that using ^11^C-PiB, has become an important tool for reliable diagnosis of Aβ plaque pathology. However, whereas amyloid plaques are a hallmark of AD, soluble Aβ may be a more dynamic marker of disease progression. Moreover, because the drugs currently in development are aimed mainly at reducing soluble Aβ, the existing amyloid PET ligands may not be optimal for quantifying the effects of treatment. In the present study, we demonstrated that a recombinantly produced PET ligand derived from the Aβ protofibril–selective antibody mAb158 can be used to visualize and quantify the progression of Aβ pathology in transgenic tg-ArcSwe mice and, further, detects changes in brain Aβ levels related to an Aβ-reducing treatment.

PET imaging requires a certain density of the target in the studied tissue to produce a signal that can be reliably quantified. Although brain concentrations of soluble Aβ protofibrils, the target of mAb158, are already elevated at 2 mo of age in tg-ArcSwe mice (27), the levels are initially low until plaques start to develop at around 6 mo of age (20), catalyzing Aβ aggregation and accumulation of soluble aggregates (28). With the PET ligand ^124^I-RmAb158-scFv8D3, Aβ pathology was already detected at the age of 7 mo, with a broad dynamic range and an increasing PET signal up to the age of 16 mo (Fig. 2; Supplemental Fig. 1A). PET results correlated well with ex vivo–measured radioactivity and Aβ pathology (Supplemental Fig. 1B; Fig. 2). This finding is to be compared with ^11^C-PiB PET imaging, which in a previous study barely detected Aβ pathology at the age of 12 mo but gave detectable signals at 18 mo (15), despite the dense, AD-like nature of tg-ArcSwe plaques (23). ^11^C-PiB, binding to the amyloid plaque core, thus seems to require a high density of plaques to produce a signal. In contrast, although dependent on the presence of plaques, ^124^I-RmAb158-scFv8D3 seems to detect a more dynamic and abundant pool of Aβ, associated with the plaques. On the basis of these findings, a study was designed to investigate the sensitivity of ^124^I-RmAb158-scFv8D3 PET to Aβ-reducing treatment at an early stage of Aβ pathology.

The reduction of Aβ accumulation in tg-ArcSwe mice was achieved with the BACE-1 inhibitor NB-360, which previously gave a prominent Aβ reduction in APP51/16 mice (21). After 3 mo of treatment, PET images obtained with the Aβ protofibril–selective radioligand ^124^I-RmAb158-scFv8D3 clearly visualized markedly reduced Aβ pathology in NB-360–treated mice in comparison with the vehicle group, similar to baseline (Fig. 3). Quantification of PET data confirmed this result, with equally low signals in baseline and NB-360–treated mice and a significantly higher signal in the vehicle group, especially in brain regions with abundant Aβ pathology, such as the cortex and hippocampus (Fig. 4). These results suggest that Aβ accumulation was essentially halted during the 3 mo of treatment and that the PET ligand clearly detected this treatment effect in vivo at a stage of pathology that is below the detection limit of ^11^C-PiB PET imaging in this animal model.

ELISA analyses of TBS brain extracts confirmed the PET results. Animals without treatment had a normal development of pathology from the age of 10 mo to the age of 13 mo, with the usual range of variation when compared with previous investigations (5,20,23). In contrast, levels of soluble Aβ aggregates (Fig. 5D), the primary target for ^124^I-RmAb158-scFv8D3, were 3-fold lower in treated mice than in their untreated littermates and were similar to the level in baseline mice. Similarly, levels of total soluble Aβ38, Aβ40, and Aβ42 were 3-fold lower in treated animals (Figs. 5A–5C), almost to the baseline level, suggesting that BACE-1 inhibition maintained low levels of soluble Aβ but did not induce an overall reduction over time. Interestingly, although not statistically significant (*P* = 0.051), the relative amount of aggregated Aβ in the soluble pool (soluble Aβ aggregates/total soluble Aβ) was lower in the treated group than in the baseline group, suggesting that the inhibited Aβ production decreased the rate of aggregation.

As a consequence of reduced production and aggregation, Aβ deposition and plaque formation were halted in NB-360–treated animals. Aβ deposits were detected with Aβ40 immunohistochemistry in the prefrontal cortex and hippocampus in the baseline group and did not change over time with NB-360 treatment. However, untreated animals with a normal course of pathology reached a significantly higher plaque load during the study (Fig. 3), as was also confirmed by ELISA quantification of total Aβ40 and Aβ42 in FA-soluble brain extracts (Figs. 5E–5F). The distinct GFAP staining around plaques seen in the vehicle group was reduced in treated mice, indicating a prominent effect of NB-360 treatment also on neuroinflammation.

Although the decreased plaque load per se could potentially contribute to the reduced PET signal observed here, previous studies with mAb158-derived ligands have shown little correlation between PET signal and insoluble Aβ. In contrast, binding was observed around plaques, and there was a good correlation with soluble Aβ protofibrils (15). Soluble Aβ aggregates have been reported to form in the area surrounding amyloid plaques (28). A decreased plaque load thus implies fewer oligomerization sites and a reduction in the formation of plaque-associated, protofibrillar Aβ. Such an Aβ reduction was previously reported after NB-360 treatment in APP23xPS45 transgenic mice (22). In the present study, ELISA analyses of postmortem brain revealed an overall reduction in soluble Aβ aggregates after BACE-1 inhibition, and notably, a smaller proportion of soluble Aβ seemed to be in an aggregated state. The substantially decreased in vivo PET signal observed here is therefore likely to represent this reduction in soluble Aβ aggregates. Altogether, these findings highlight potential for measuring soluble Aβ aggregates in vivo to monitor disease progression and detect effects of Aβ reducing treatment. These findings may also explain the pronounced difference in PET signal achieved with ^124^I-RmAb158-scFv8D3 after BACE-1 inhibition, as compared with the modest differences detected with the small-molecule PET ligand ^18^F-florbetapir (13), which visualizes the dense core of amyloid plaques.

## CONCLUSION

Antibody-based PET imaging of soluble Aβ protofibrils is a sensitive tool for following progression of brain Aβ pathology and the treatment effects achieved by inhibition of Aβ production. This study is a step toward a method that might be used in future preclinical and clinical studies of novel AD drug candidates.

## DISCLOSURE

Ulf Neumann is an employee and shareholder of Novartis Pharma AG, Basel, Switzerland. Lars Lannfelt is a founder and shareholder of BioArctic AB, Stockholm, Sweden. Financial support was granted from the Swedish Research Council (grant 2017-02413), Alzheimerfonden, Hjärnfonden, Torsten Söderbergs stiftelse, Hedlunds stiftelse, Stiftelsen Fondkistan, Åhlén-stiftelsen, Stiftelsen Sigurd och Elsa Goljes minne, Stohnes stiftelse, Stiftelsen för Gamla tjänarinnor, Magnus Bergwalls stiftelse, and the Uppsala Berzelii Technology Centre for Neurodiagnostics. The molecular imaging work in this study was performed at the SciLifeLab Pilot Facility for Preclinical PET-MRI, a Swedish nationally available imaging platform at Uppsala University, Sweden, financed by the Knut and Alice Wallenberg Foundation. No other potential conflict of interest relevant to this article was reported.
