# Supplementary material for: Antibody-Based In Vivo PET Imaging Detects Amyloid-β Reduction in Alzheimer Transgenic Mice After BACE-1 Inhibition
Source: J Nucl Med. 2018 Dec;59(12):1885–91. doi: 10.2967/jnumed.118.213140 (PMC6278900; doi:10.2967/jnumed.118.213140)
Supplement: Supplementary file 1 [file jnm213140SupplementalData.pdf]

Supplemental Table 1: Animal Groups

Group		***Age***	***Number of animals***
7 month	Disease progression study	7m	n=2
10 month	Disease progression study	10m	n=2
13 month	Disease progression study	13m	n=2
16 month	Disease progression study	16m	n=3
Baseline	Treatment study, baseline group (analyzed at starting age)	10m	n=9
NB-360	Treatment study, treated group (with BACE-1 inhibitor)	13m	n=9
Vehicle	Treatment study, control group (without BACE-1 inhibitor)	13m	n=11

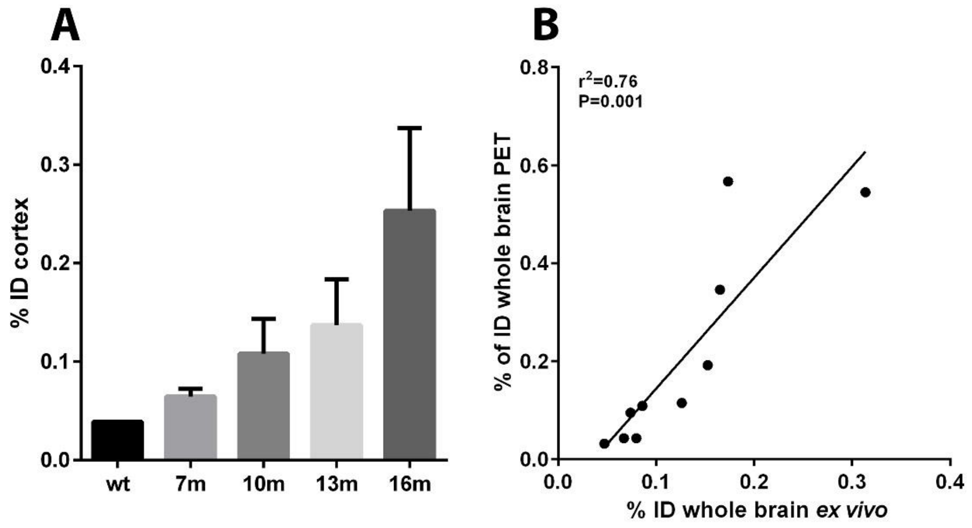

Supplemental Figure 1. PET quantifiaction of disease progression and comparsion between PET and *ex vivo* activity. A. PET image-based quantification of radioactivity, expressed as %ID per gram brain tissue, in cortex of ArcSwe animals at the age of 7, 10, 13, and 16 month in comparison with old wildtype mice. **B.** Correlation of % of injected dose between whole brain PET and *ex vivo* measurements.
